# Mff‐Dependent Mitochondrial Fission Contributes to the Pathogenesis of Cardiac Microvasculature Ischemia/Reperfusion Injury via Induction of mROS‐Mediated Cardiolipin Oxidation and HK2/VDAC1 Disassociation‐Involved mPTP Opening

**DOI:** 10.1161/JAHA.116.005328

**Published:** 2017-03-13

**Authors:** Hao Zhou, Shunying Hu, Qinhua Jin, Chen Shi, Ying Zhang, Pingjun Zhu, Qiang Ma, Feng Tian, Yundai Chen

**Affiliations:** ^1^ Department of Cardiology Chinese PLA General Hospital Beijing China; ^2^ Department of Radiation Oncology Peking University Cancer Hospital and Institute Beijing China

**Keywords:** apoptosis, endothelial cell, ischemia/reperfusion injury, mitochondria, Basic Science Research, Cell Biology/Structural Biology, Coronary Circulation, Endothelium/Vascular Type/Nitric Oxide, Vascular Biology

## Abstract

**Background:**

The cardiac microvascular system ischemia/reperfusion injury following percutaneous coronary intervention is a clinical thorny problem. This study explores the mechanisms by which ischemia/reperfusion injury induces cardiac microcirculation collapse.

**Methods and Results:**

In wild‐type mice, mitochondrial fission factor (Mff) expression increased in response to acute microvascular ischemia/reperfusion injury. Compared with wild‐type mice, homozygous Mff‐deficient (Mff^gt^) mice exhibited a smaller infarcted area, restored cardiac function, improved blood flow, and reduced microcirculation perfusion defects. Histopathology analysis demonstrated that cardiac microcirculation endothelial cells (CMECs) in Mff^gt^ mice had an intact endothelial barrier, recovered phospho‐endothelial nitric oxide synthase production, opened lumen, undivided mitochondrial structures, and less CMEC death. In vitro, Mff‐deficient CMECs (derived from Mff^gt^ mice or Mff small interfering RNA–treated) demonstrated less mitochondrial fission and mitochondrial‐dependent apoptosis compared with cells derived from wild‐type mice. The loss of Mff inhibited mitochondrial permeability transition pore opening via blocking the oligomerization of voltage‐dependent anion channel 1 and subsequent hexokinase 2 separation from mitochondria. Moreover, Mff deficiency reduced the cyt‐c leakage into the cytoplasm by alleviating cardiolipin oxidation resulting from damage to the electron transport chain complexes and mitochondrial reactive oxygen species overproduction.

**Conclusions:**

This evidence clearly illustrates that microcirculatory ischemia/reperfusion injury can be attributed to Mff‐dependent mitochondrial fission via voltage‐dependent anion channel 1/hexokinase 2–mediated mitochondrial permeability transition pore opening and mitochondrial reactive oxygen species/cardiolipin involved cyt‐c release.

## Introduction

Prompt myocardial reperfusion through timely percutaneous coronary intervention is the most effective therapeutic approach for reducing infarct size and improving clinical outcome following acute myocardial infarction. Nevertheless, despite successful revascularization of the epicardial occluded vessels, the reperfusion failure at the microvascular bed postischemia, termed microcirculatory ischemia/reperfusion (IR) injury^1^, ^2^ occurs in ≈30% of patients with acute myocardial infarction.^3^, ^4^ Therefore, an understanding of microvascular IR injury is essential in order to prevent microcirculation IR injury as well as to design strategies aimed at improving microcirculatory damage.

The mitochondrion is the center of energy and metabolism of eukaryotes that acts by generating ATP. It is also the key regulator of programmed cell death pathways and has crucial functions in multiple signaling pathways. During IR injury, mitochondria in cardiomyocytes induce cellular damage due to their inability to supply energy and their generation of excessive reactive oxygen species. However, cardiac microcirculation endothelial cells (CMECs) have few mitochondria; for example, in rats, endothelial mitochondria compose 2% to 6% of the cell volume, as opposed to 32% in cardiac myocytes.^5^ Furthermore, 99% of glucose is metabolized to lactate via anaerobic respiration in isolated coronary microvascular endothelial cells.^6^, ^7^ These data indicate that the mitochondria in endothelial cells are not solely responsible for energy metabolism. In the vascular endothelium, mitochondria are more likely to serve primarily as essential cellular apoptosis signaling organelles. However, little is understood regarding the exact role of mitochondria in microcirculatory IR injury and in the apoptosis of CMECs. Importantly, mitochondria are not static organelles; instead, they move and continuously change their appearance by fission, fusion, and branching. To ensure organelle integrity, mitochondria constantly fuse and divide to generate functionally normal mitochondria through a process called mitochondrial dynamics. Through modest mitochondrial fission, dysfunctional mitochondria are separated from the integrated network to maintain organelle quality and homeostasis. However, whether microvascular IR injury is associated with excessive mitochondrial fission remains unknown.

Several studies have focused on the important role of dynamin‐related protein 1 (Drp1) during fission. This protein has been reported to redistribute from a predominately cytosolic location to predicted sites of division along mitochondrial tubules.^8^ In fact, Drp1 recruitment to the mitochondria is dependent on its corresponding receptors, mitochondrial fission 1, and mitochondrial fission factor (Mff), which are located on the mitochondrial outer membrane. These receptors tightly couple cytoplasmic Drp1 to the mitochondria.^9^ Otera et al^10^, ^11^ provided experimental evidence that Mff and not mitochondrial fission 1 preferentially captures Drp1, suggesting that Mff is required for successive fission. Their data also strongly highlight the neglected but critical information regarding fission because the Drp1 receptor Mff has been substantially underestimated by many reports when compared with Drp1 and mitochondrial fission 1. As a consequence of fission, cyt‐c leakage via the mitochondrial permeability transition pore (mPTP) occurs and subsequently initiates mitochondrial‐dependent cellular apoptosis in cardiomyocytes. However, the mechanism by which fission induces mPTP opening and cyt‐c release remains unclear. Hexokinase 2 (HK2) is a rate‐limiting enzyme of cytoplasm anaerobic respiration in CMECs, which also anchors to the outer mitochondrial membrane via interaction with voltage‐dependent anion channel 1 (VDAC1), contributing to the inhibition of excessive mPTP opening by structurally interfering with the ability of proapoptotic proteins such as B‐cell lymphoma 2–associated X (Bax) and B‐cell lymphoma 2–associated death promoter (Bad) to translocate from the cytoplasm and bind to VDAC1.^12^ Additionally, under physiological conditions, cyt‐c is trapped tightly within the inner mitochondrial membrane by cardiolipin (CL).^13^ The selective peroxidation of CL facilitates the detachment of cyt‐c from the outer surface of the inner mitochondrial membrane and its subsequent release into the cytoplasm via the mPTP.^14^ However, it remains unclear whether Mff‐associated fission triggers mPTP opening and cyt‐c leakage via the regulation of HK2 disassociation and CL oxidation and, if so, which molecule links fission to the changes in HK2 liberation and CL peroxidation. In the present study, we explored the relationship between Mff‐mediated fission and cardiac microvascular IR injury via homozygous Mff‐deficient (Mff^gt^) mice and in vitro gain‐ and loss‐of‐function experiments.

## Methods

### Animal and Ex Vivo Models of Cardiac IR Injury

All protocols were approved by the PLA General Hospital Institutional Animal Care and Use Committee. We generated Mff‐deficient mice with a homozygous gene trap of Mff according to methods described in previous studies.^15^ The Mff gene trap embryonic stem cell clone was obtained from Cam‐Su Genomic Resource Center (Soochow University, China) and was microinjected into mouse blastocysts by the Cam‐Su Genomic Resource Center. In brief, the exon/intron structure was derived from Ensembl and the gene trap vector pGT01xr with a splice acceptor and lacZ/neomycin phosphotransferase fusion gene (β‐geo) was inserted immediately after the sequence 5′‐GCACTCCTCTGTCTGCCTTG‐3′ in the intron following exon 4 (Figure S1) according to the reports by Chen et al.^15^ The mice were generated on a C57BL/6 background. Mff^gt^ and wild‐type (WT) male mice (6–8 weeks old) were used to induce an IR injury model. Mice were anesthetized with sodium pentobarbital (50 mg/kg IP) and ketamine hydrochloride (50 mg/kg IP). Then, a 7‐0 silk suture was passed underneath the left anterior descending coronary artery and a slipknot was tied with 30 minutes of ischemia followed by 2 hours of reperfusion. At the end of the reperfusion period, the hearts were stained with 2% Evans Blue and 1% 2,3,5‐triphenyltetrazolium chloride. The infarct size was expressed as a percentage of the risk zone as previously described (n=6/group).

### CMEC Culture, Identification, Experiments Group, and IR Injury Induction In Vitro

CMECs were isolated using the enzyme dissociation method and identified by CD31 staining and uptake of acetylated low‐density lipoprotein as previously described.^16^ In vitro, CMECs obtained from WT and Mff^gt^ mice were classified into WT and Mff^gt^ groups, respectively. Meanwhile, Mff loss‐ and gain‐of‐function experiments were performed by Mff small interfering RNA (WT+RNAi_‐Mff_ group) and Mff adenovirus vector overexpression (Ad+Mff^gt^ group). The IR injury in vitro was mimicked by 30 minutes of hypoxia with serum starvation and 2 hours of reoxygeneration. Hypoxic conditions used fresh Hanks solution with 95% N_2_ and 5% CO_2_. The pH was adjusted to 6.8 with lactate to mimic ischemic conditions.

### Echocardiogram and Microvascular Imaging by Gelatin‐Ink Perfusion

Echocardiography was performed in all mice after 2 hours of reperfusion. Mice (n=6) were anaesthetized with intraperitoneal injections of sodium pentobarbital (50 mg/kg) and ketamine hydrochloride (50 mg/kg) for function measurement with echocardiogram (14.0 MHz, Sequoia C512; Acuson, Germany). Both 2‐dimensional and M‐mode images were recorded.

Gelatin‐ink perfusion was conducted after IR injury. The 37°C ink plus 3% gelatin (gelatin‐ink staining) was perfused via the jugular vein and kept at a room temperature of 25 to 30°C. When the limbs turned black, the great vessels of the cardiac base and the superior and inferior vena cava were ligated. The hearts were subsequently maintained at 4°C for at least 1 hour, then removed and fixed in 4% paraformaldehyde, and processed for cryosectioning.

### Immunohistochemistry, Immunofluorescence Staining, Cross‐Linking of VDAC1

Immunohistochemical staining was conducted on 4‐mm sections of heart tissue using Mff 1:500 (Abcam), phospho‐endothelial nitric oxide synthase (p‐eNOS; Ser1177) 1:200 (Abcam), intercellular adhesion molecule–1 1:500 (Abcam), vascular cell adhesion molecule–1 1:500 (Abcam), F4/80 1:500 (Abcam), and plasma albumin 1:500 (Abcam). The primary antibodies were as follows: CD31 (1:1500, Abcam), vascular endothelial cadherin (VE‐cadherin; 1:1000, Abcam), cyt‐c (1:500, Abcam), Mff (1:1000, Abcam), pDrp1 (1:500, Abcam), HK2 (1:500, Abcam), cleaved caspase3 (CL.caspase3; 1:1000, Abcam), and pro‐caspase3 (1:2000, Abcam). 4′,6‐Diamidino‐2‐phenylindole dihydrochloride (Sigma‐Aldrich, USA), lysosome stain, and a mitochondrion‐selective MitoFluor stain (Molecular Probes, USA) were used to marker the nuclear, lysosome, and mitochondria, respectively. For the cross‐linking of VDAC1, treated cells were harvested and added with dimethyl sulfoxide as a vehicle control (2%, as used in compound‐containing samples) or cross‐linked with 0.5 mm ethylene glycolbis (succinimidyl succinate) for 10 minutes at 30°C followed by 20 mm Tris‐HCl (pH 7.4) to quench the reaction. Samples were determined by SDS‐PAGE via immunoblotting.

### Mitochondrial Morphology Analysis

The change of mitochondrial morphology was conducted via confocal microscope. After cells were fixed in 4% paraformaldehyde, TOM20 primary antibody was used to label the mitochondria. Then the single‐cell image was obtained, which was analyzed using ImageJ 1.47 version software according to previous studies.^17^

### mROS, NAO Staining, and Mitochondrial Membrane Potential Measurements

Mitochondrial reactive oxygen species (mROS) measurement was conducted using a MitoSOX red mitochondrial superoxide indicator (Molecular Probes, USA). Staining with 10‐N‐nonyl acridine orange (NAO; 2 mmol/L, Molecular Probes) was used. The mitochondrial transmembrane potential was analyzed using a JC‐1 Kit (Beyotime, China). Images were captured using a fluorescence microscope (OLYMPUS DX51; Olympus, Tokyo, Japan) and were analyzed with Image‐Pro Plus 6.0 (Media Cybernetics, Rockville, MD) to obtain the mean densities of the region of interest, which was normalized to that of the control group.

### Apoptosis Detection, mPTP Opening, and ATP Production

terminal deoxynucleotidyl transferase dUTP nick‐end labeling assay (Roche Applied Bio Sciences, USA) was used to assess the cellular apoptosis following the instructions. For quantification, the number of terminal deoxynucleotidyl transferase dUTP nick‐end labeling–positive cells was calculated by counting at least 5 random separate fields as the ratio of the experimental samples to the control samples.

The opening of the mPTP was visualized as a rapid dissipation of tetramethylrhodamine ethyl ester fluorescence. Arbitrary mPTP opening time was determined as the time when tetramethylrhodamine ethyl ester fluorescence intensity decreased by half between initial and residual fluorescence intensity according to previous study.^18^ The cellular ATP levels were measured using a firefly luciferase‐based ATP assay kit (Beyotime) based on a fluorescence technique (Genmed Scientifics Inc.) according to the specified protocol.

### Western Blot, Coimmunoprecipitation, and Electron Microscopy

Equal amounts of proteins were loaded on 8% to 15% SDS‐PAGE gels and then transferred to PVDF membranes (Sigma‐Aldrich, USA). After blocked with 5% skim milk for 90 minutes at room temperature, the sheets were incubated with primary antibodies at 4°C overnight (cyt‐c [1:500], cleaved caspase‐3 [1:1000], caspase‐9 [1:1000], myeloid cell leukemia 1 [1:1000], VDAC1 [1:1500], pDrp1 [1:500], and HK2 [1:1000] were purchased from Abcam; X‐linked inhibitor of apoptosis protein [1:1000], B‐cell lymphoma 2 [1:2000], Mff [1:500], mitofusin 1 [1:2000], and mitofusin 2 [1:2000] were purchased from Cell Signaling Technology). Furthermore, the mitochondrial oxidative phosphorylation proteins were semiquantified using a MitoProfile Total OXPHOS Western blotting kit (1:1000 mouse monoclonal IgG; Abcam), which contained a cocktail of monoclonal antibodies: complex III subunit core 2, 47 kDa—“CIII‐core2”; complex II‐FeS subunit, 30 kDa—“CII‐30”; complex IV subunit II, 24 kDa—“CIV‐II”; and complex I subunit NDUFB8, 20 kDa—“CI‐20”.

Coimmunoprecipitation experiments were performed as already described. Briefly, proteins from cells were cross‐linked in 1% paraformaldehyde followed by washing in PBS containing 100 mmol/L glycine. The cells were then lysed by sonication in PBS with 1% Triton X‐100 and incubated with the respective antibodies and protein A/G agarose. The immunoprecipitates were loaded on SDS‐PAGE and probed with HK2 antibody, as described above. The mean densities of the bands in the blots were measured and normalized to that of β‐actin (Quantity One, version 4.6.2).

### Electron Microscopy

After treatment, cells and tissues were immediately fixed at 4°C with 2% glutaraldehyde in 0.1 mol/L sodium cacodylate buffer and postfixed for 1 hour on ice with 1% osmium tetroxide. The cells and tissues were rinsed with distilled water and dehydrated using acetonitrile and graded methanol (50%, 20 minutes; 70%, 20 minutes; 95%, 20 minutes; and 100% 3×, 20 minutes), and then were embedded in epoxy resin (EMbed‐812; Electron Microscopy Sciences, USA) and polymerized at 70°C overnight. Thin (60 nm) sections were cut, and the sections were stained with lead citrate and uranyl acetate. The samples were imaged using a Hitachi H600 Electron Microscope (Hitachi, Japan). At least 30 cells in at least 5 randomly selected fields were observed.

### mtDNA Strand Breaks, Copy Numbers, Transcription Level Detection, and Respiratory Chain Complex Activities Assays

mtDNA strand breaks were detected based on methods described in previous studies.^19^ Briefly, a 200‐μL mitochondrial suspension was centrifuged at 15 000*g* at 4°C for 20 minutes. After the supernatant was discarded, a 400‐μL solution (0.25 mmol/L inositol, 10 mmol/L Na_3_PO_4_, and 1 mmol/L MgCl_2_, pH 7.2) was added at 4°C for 30 minutes. The following steps were performed according to the fluorometric analysis of DNA unwinding methods reported by Birnboim and Jevcak.^19^ The data are expressed as the percentage of double‐stranded DNA.

The relative amounts of mtDNA and nDNA content were used to assess the mtDNA copy numbers via reverse transcription polymerase chain reaction. The mtDNA and nuclear amplicons were generated from a complex IV segment and GAPDH segment, respectively. The mtDNA primers were 5′‐CCCCTGCTATAACCCAATACA‐3′ and 5′‐CCAAACCCTGGAAGAATTAAGA‐3′ (fragment length, 238 bp). The GAPDH primers, chosen as the internal standards, were 5′‐TGTTGCTGTAGCCATATTCATTGT‐3′ and 5′‐CCATTCTTCCACCTTTGATGCT‐3′ (fragment length, 98 bp).

The transcript level of mtDNA was reflected by two different components: NADH dehydrogenase subunit 1 (ND1), which is encoded by the light chain of mtDNA, and cytochrome c oxidase subunit I, which is encoded by the heavy chain. The primers for cytochrome c oxidase subunit I were 5′‐CCCCTGCTATAACCCAATACA‐3′ and 5′‐CCAAACCCTGGAAGAATTAAGA‐3′ (fragment length, 238 bp). The primers for ND1 were 5′‐CGGCTCCTTCTCCCTACAA‐3′ and 5′‐ATGGTCCTGCGGCGTATT‐3′ (fragment length, 188 bp). GAPDH was selected as the internal standard. The experiments were repeated 3 times with triplicates of each sample. The relative mRNA expression levels were normalized to that of GAPDH using the 2^−ΔΔCT^ method according to our previous study.^16^

Complex I, II, and V activity was measured according to previous studies.^20^ Mitochondrial respiratory function was measured polarographically at 30°C using a Biological Oxyge Monitor System (Hansatech Instruments, King's Lynn, UK) and a Clarktype oxygen electrode (Hansatech DW1, Norfolk, UK). Mitochondrial respiration was initiated by adding glutamate/malate to a final concentration of 5 and 2.5 mmol/L, respectively. State 3 respiration was initiated by adding ADP (150 nmol/L); state 4 was measured as the rate of oxygen consumption after ADP phosphorylation. The respiratory control ratio (state 3/state 4) and the ADP/O ratio (number of nmol ADP phosphorylated to atoms of oxygen consumed) were calculated as previously described.^21^

### Measurement of TER and Permeability of CMECs

Transendothelial electrical resistance (TER) is a measure of the ionic conductance of endothelial cells and could be used to assess junctional function.^22^ TER decreases when endothelial cells retract or lose adhesion. With the In Vitro Vascular Permeability Assay kit (ECM640, Millipore, USA), CMECs at 100 000 cells per insert were seeded onto collagen‐coated inserts. After confluence, the cells were subjected to I/R and an electrical endothelial resistance system (Millipore, USA) was used to measure TER as previously described.^23^ Fluorescein isothiocyanate (FITC)–dextran clearance was measured to assess changes in endothelial permeability. FITC‐dextran (final concentration 1 mg/mL) was added on top of the inserts, allowing it to permeate through the cell monolayer. The extent of permeability after 2 hours was determined by using a fluorescent plate reader (Bio‐Rad, USA) to measure the FITC content remaining on the plate.

### Construction of Adenovirus for Mff Overexpression

The plasmid of pDC316‐mCMV‐Mff was purchased from Vigene Bioscience and transfected with framework plasmid (1:1) into 293 T cells by using lipofectamine 2000. After transfection for 48 hours, the viral supernatant was collected and identified by polymerase chain reaction. After amplification, the supernatant was acquired again and filtered through a 0.45‐μm filter to obtain the Ad‐Mff. Then the final supernatants were used to infect CMCEs for 4 hours at 37°C/5% CO_2_ with continuous shaking. The media was then replaced with fresh culture medium with 20% FBS. After 24‐hour culture, the cells were used to evaluate the expression of Mff with Western blots, and the results of transfection efficiency are shown in Figure S2A and S2B.

### CL Extraction and High‐Performance Thin‐Layer Chromatography Analysis

CMEC mitochondrial homogenates were used to obtain CL by and high‐performance thin‐layer chromatography with an electrospray ionization source and a linear ion trap mass spectrometer (LXQ Thermo‐Fisher). CL and its oxidized molecular species were extracted using the Folch procedure^24^ and separated on a normal phase column (Luna 3 μm Silica 100 A, 150×2 mm; Phenomenex, Torrance, CA) with a flow rate of 0.2 mL/min and the application of a gradient elution using solvents containing 5 mmol/L CH_3_COONH_4_ (A‐n‐hexane:2‐propanol:water, 43:57:1 [v/v/v] and B‐n‐hexane:2‐propanol:water, 43:57:10 [v/v/v]). The analysis of (hydroperoxy‐ and hydroxy‐) oxidized phospholipid species was performed as previously described.^25^

### Statistical Analysis

All data are represented as mean±SEM. The differences among more than 2 groups were evaluated through 1‐way ANOVA (all data met the variances homogeneity and normal distribution). The multiple comparison procedure was used to compare the statistical difference between 2 groups. A value of *P*<0.05 was considered statistically significant. All of the statistical analyses were performed with SPSS for Windows version 16.0 (SPSS Inc., Chicago, IL).

## Results

### Mff is Upregulated in IR Injury, Contributing to Infarct Size Expansion and Cardiac Dysfunction

**Figure 1:**
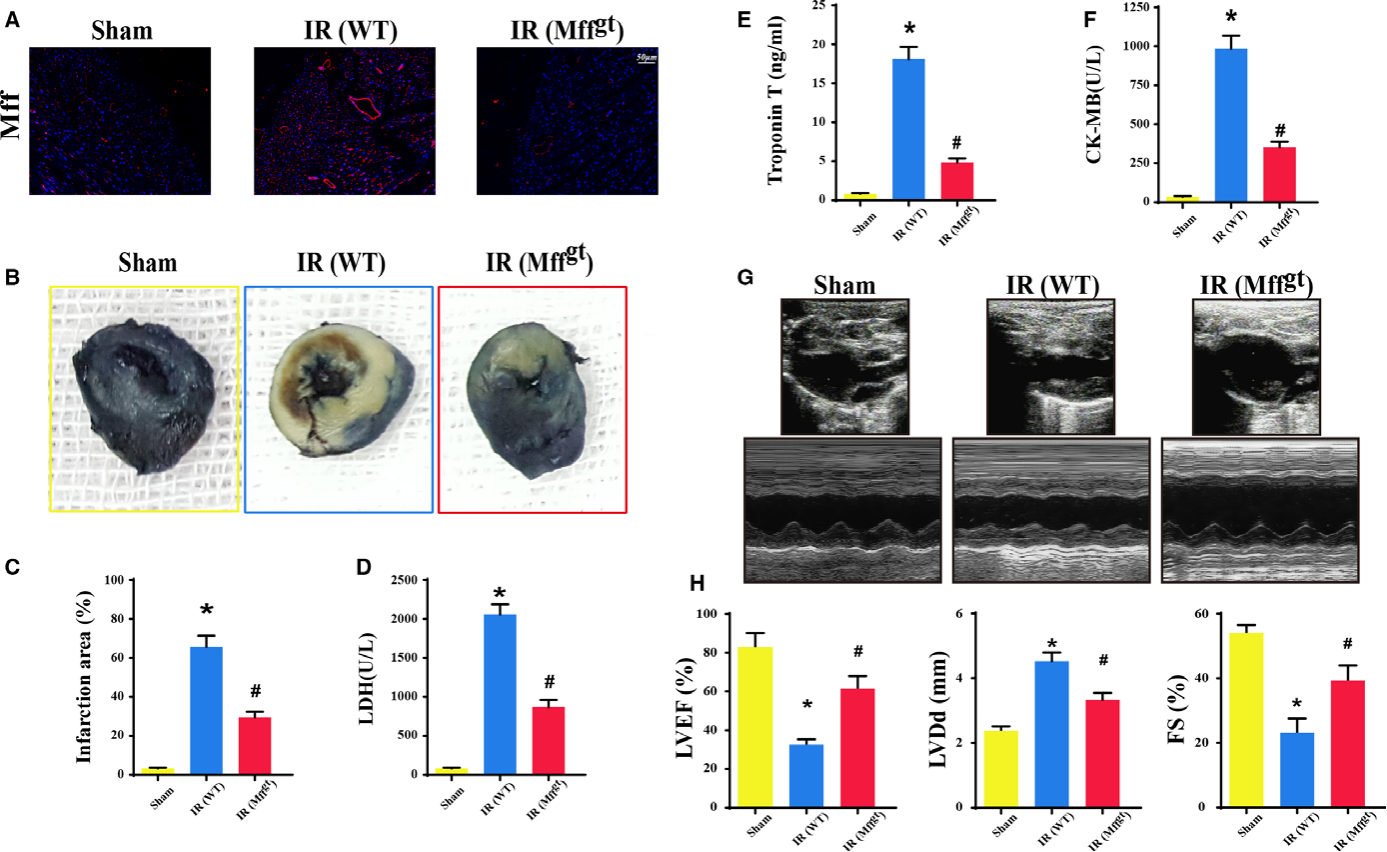
Mitochondrial fission factor (Mff) contributed to infarct size expansion and cardiac function deterioration following ischemia/reperfusion (IR) injury in vivo, which was performed by 30 minutes of ischemia followed by 2 hours of reperfusion (n=6/group). A, Upregulation of Mff in microvascular in response to IR injury by immunofluorescence. B, Representative pictures of heart sections with 2,3,5‐triphenyltetrazolium chloride and Evans Blue staining. C, Bar graph indicates the infarct size expressed as a percentage of the total left ventricular area. D through F, The lactate dehydrogenase (LDH) release, Troponin T contents, and creatine kinase‐MB (CK‐MB) values were assessed via ELISA. G, Representative M‐mode echocardiography was performed after 2 hours of reperfusion with the parasternal long‐axis views in each group. H, Quantitative analysis of cardiac function by echocardiography. *P<0.05 vs the sham group; ^#^ *P*<0.05 vs the wild‐type (WT) group. FS indicates fractional shortening; LVDd, left ventricular diastolic dimension; LVEF, left ventricular ejection fraction; Mff^gt^, homozygous Mff‐deficient mice.

To investigate the physiological role of Mff in vivo, we initially assessed Mff expression before and after IR injury in WT mice. Compared with baseline, IR induced a significant increase in Mff protein expression in WT mice but not in Mff^gt^ mice (Figure 1A) via the immunofluorescence analysis. Next, to explore the role of Mff in acute microvascular IR injury, the infarct size was observed. As shown in Figure 1B and 1C, the infarct size, which was expressed as the percentage of the left ventricle, was significantly decreased in the Mff^gt^ mice compared with the WT mice. Furthermore, lactate dehydrogenase, Troponin T, and creatine kinase‐MB expression (Figure 1D through 1F) were obviously increased in WT mice but decreased in Mff^gt^ mice. Compared with the IR group, left ventricular ejection fraction, left ventricular diastolic dimension, and left ventricular fractional shortening were significantly increased in the Mff^gt^ group (Figure 1G and 1H).

### Reduced Perfusion Defects and Microvessel Malfunction in Mff^gt^ Mice

**Figure 2:**
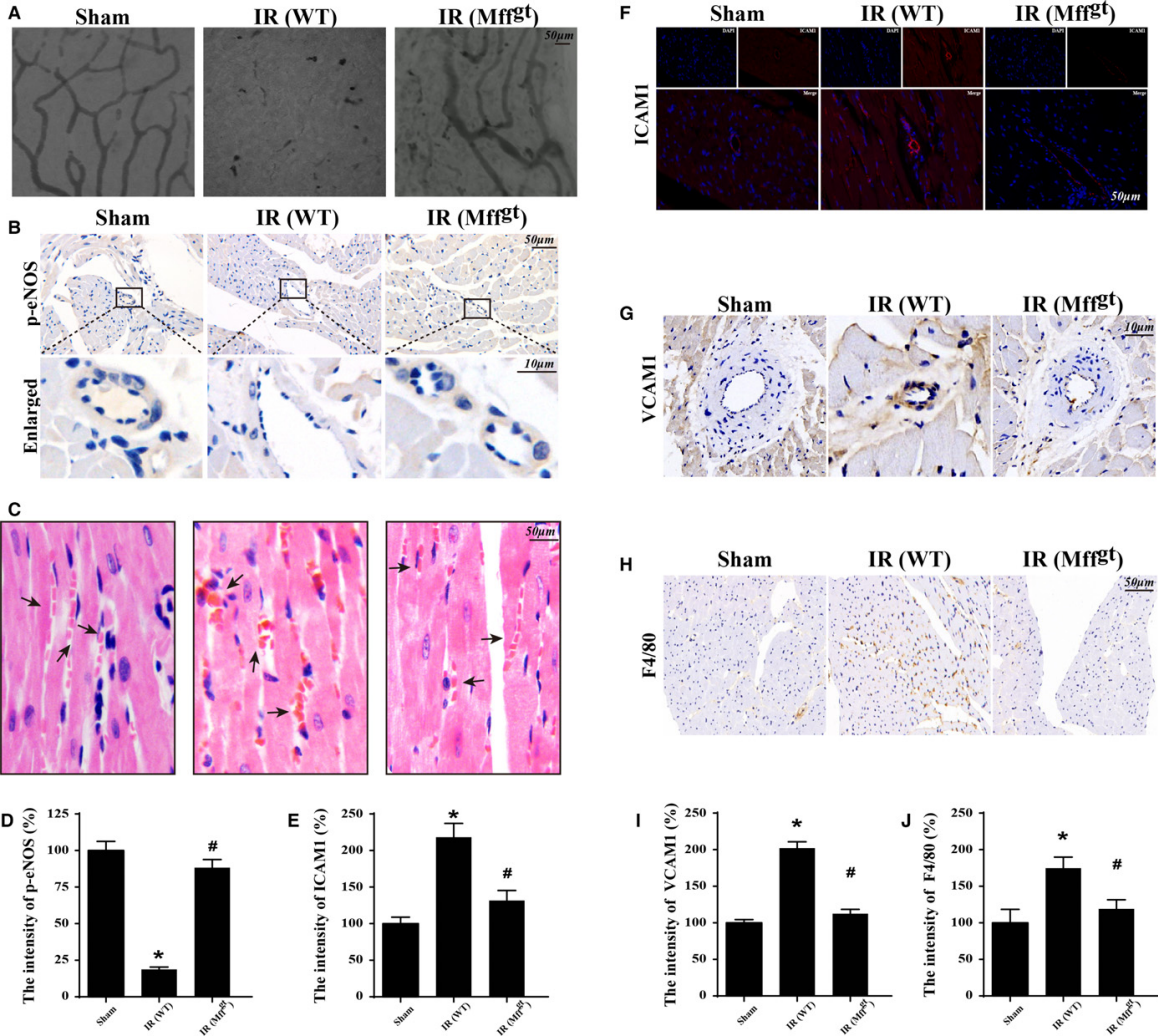
Mitochondrial fission factor (Mff) was involved in acute microcirculation malfunction during ischemia/reperfusion (IR) injury. A, Microvascular image detection by ink staining. B and D, Immunohistochemistry of phosphoendothelial nitric oxide synthase (p‐eNOS) expression. C, Hematoxylin and eosin staining for red blood cell morphology in different groups. E and F, The change of intercellular adhesion molecule–1 (ICAM1) expression in response to IR with or without Mff. G and I, Reduced vascular cell adhesion molecule–1 (VCAM1) contents under IR unless loss of Mff. H and J, Representative images of the accumulation of F4/80^+^ in myocardial tissue. *P<0.05 vs the sham group; ^#^ *P*<0.05 vs the wild‐type (WT) group. Mff^gt^ indicates homozygous Mff‐deficient mice.

Perfusion defect was the feature of microcirculation IR injury, which aggravated the deterioration of cardiac function. Gelatin ink was used to assess the microvascular perfusion defect following IR injury. IR injury induced the bloodstream delay or stop, whereas the Mff^gt^ mice recovered the patency of the microvascular blood flow (Figure 2A). Considering microvascular patency was dependent on endothelial nitric oxide synthase–dependent dilation, we observed p‐eNOS expression on microvessels. IR substantially reduced the contents of p‐eNOS, whereas the depletion of Mff increased the p‐eNOS levels (Figure 2B and 2D). Moreover, IR induced the morphological change of red blood cells from “parachute” or “arrow” to “swollen” or “massed” due to the stop of turbulent blood flow or the secondary effect owing to the capillary blockage by other cells such as leukocytes (Figure 2C). However, red blood cells in the Mff^gt^ mice exhibited a regular shape. Furthermore, IR could increase intercellular adhesion molecule–1 (Figure 2E and 2F) and vascular cell adhesion molecule–1 (Figure 2G and 2I) expression on the surface of microvascular but loss of Mff could reduce the contents of adhesive protein expressed on the surface of CMECs. Furthermore, IR also induced more F4/80^+^ cells accumulation in the myocardial tissues, which was reversed by loss of Mff (Figure 2H and 2J).

### Loss of Mff Improves Microvascular Mitochondrial Structure, the Endothelial Barrier, and CMEC Survival in IR Injury

**Figure 3:**
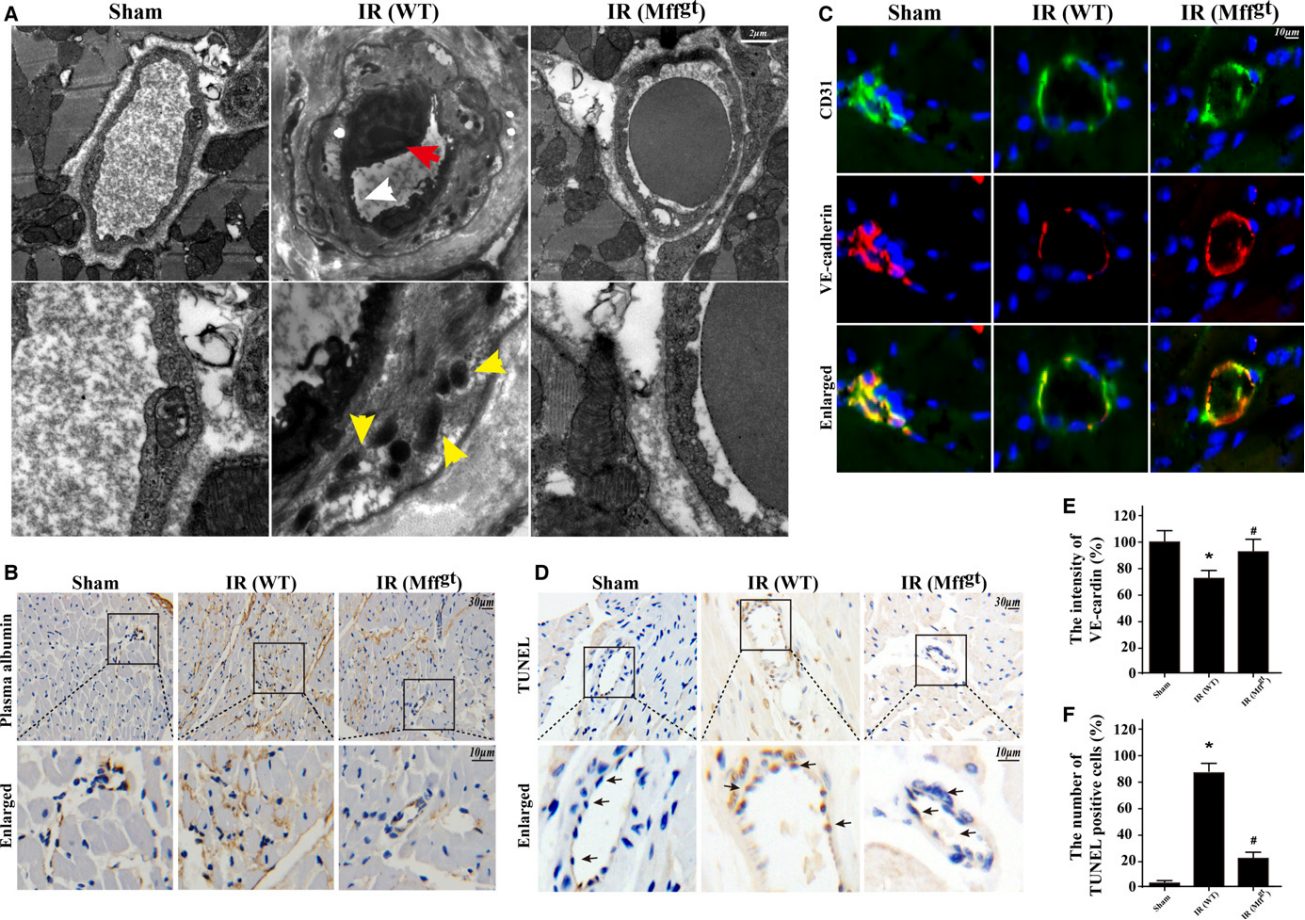
Loss of mitochondrial fission factor (Mff) maintained microvessels lumen patency, preserved endothelial barrier integrity, reduced vascular permeability, sustained cardiac microcirculation endothelial cell (CMEC) mitochondrial structure, and alleviated cellular death. A, Transmission electron microscopy was used to observe the structural changes of microvessel in response to ischemia/reperfusion (IR) injury, including microvascular wall destruction (white arrow), luminal stenosis (red arrow), and mitochondrial damage (yellow arrow) of CMEC. B, The leakage of plasma albumin out of the surface of the vessel wall into interstitial spaces suggested the increased microvascular permeability in response to after IR injury. C and E, The endothelial barrier integrity was assessed via vascular endothelial cadherin (VE‐cadherin) staining. Discontinuous punctiform or linear expression of VE‐cadherin could be observed in the IR injury group indicative of the broken endothelial barrier. However, loss of Mff could reverse the continuous linear of VE‐cadherin fluorescence. D and F, terminal deoxynucleotidyl transferase dUTP nick‐end labeling (TUNEL) assay to assess microvascular apoptosis. *P<0.05 vs the sham group; ^#^ *P*<0.05 vs the wild‐type (WT) group. Mff^gt^ indicates homozygous Mff‐deficient mice; WT, wild‐type.

Considering the role of Mff in mitochondrial fission, we observed changes in the microvascular mitochondria. In Figure 3A, irregular endothelial swelling and luminal stenosis were identified in the cardiac microvessels following IR injury in mice. Furthermore, more ruptured mitochondria or vacuolated mitochondria were identified in the IR groups. However, the surface of the cardiac microvessels was smooth and well‐integrated in Mff^gt^ mice. IR injury also induced more plasma albumin leakage from the vessel, which was confined within the blood vessel lumen until the collapse of the endothelial barrier and the diffusion of the plasma into the outer surface of the vessel wall (Figure 3B). Healthy endothelia have the ability to form the microvascular barrier through VE‐cadherin proteins (Figure 3C and 3E). However, IR injury markedly decreased the expression of VE‐cadherin, which was restored by the loss of Mff. Finally, a terminal deoxynucleotidyl transferase dUTP nick‐end labeling assay (Figure 3D and 3F) also suggested that Mff was involved in endothelial integrity and CMEC apoptosis.

### Mff Induces CMECs Apoptosis and Barrier Dysfunction via Excessive Mitochondrial Fission

**Figure 4:**
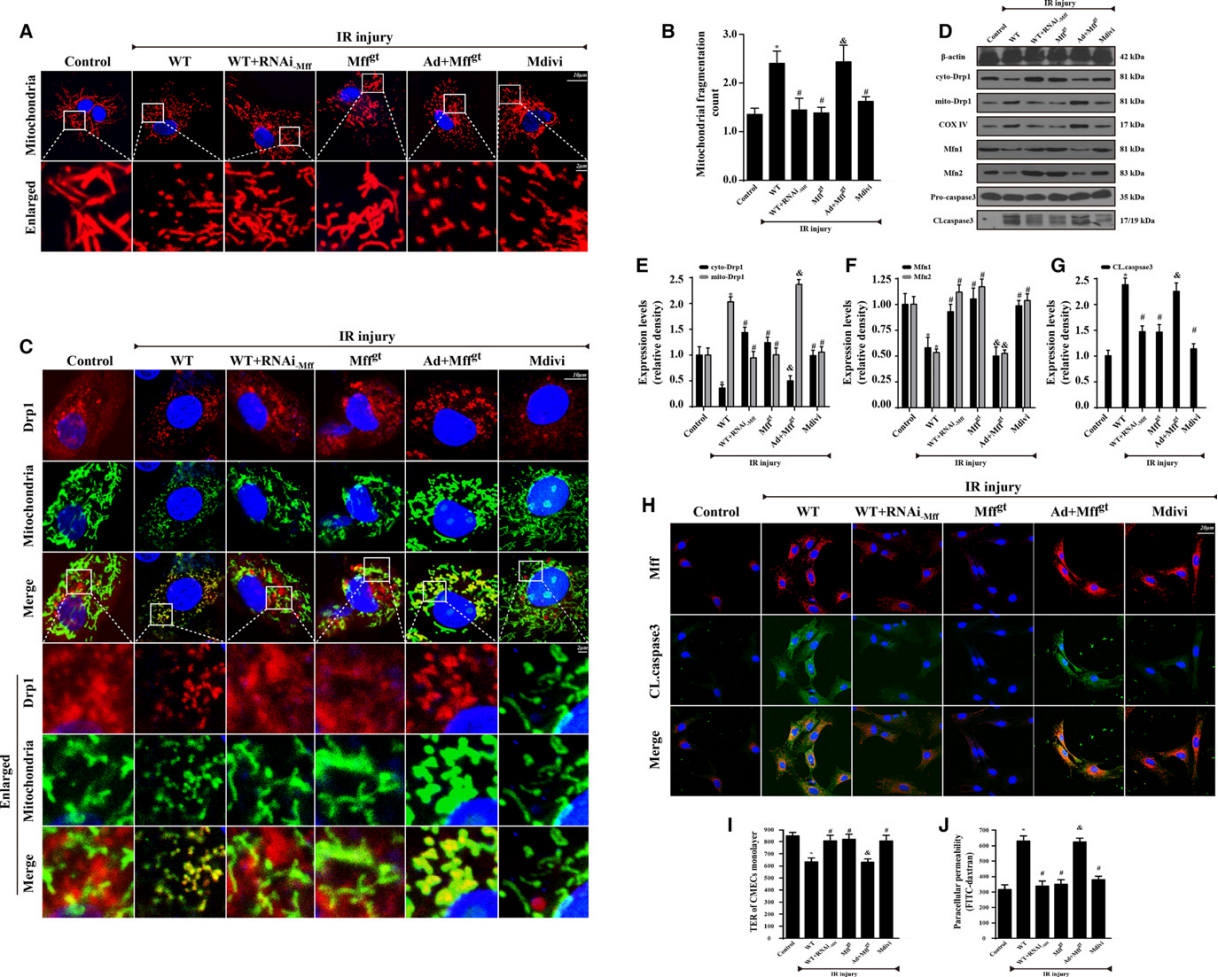
Mitochondrial fission factor (Mff) induced cardiac microcirculation endothelial cells (CMECs) apoptosis via excessive mitochondrial fission in vitro. Wild‐type (WT) mice– and homozygous Mff–deficient (Mff^gt^) mice–derived CMECs were named WT and Mff^gt^ groups, respectively. Furthermore, Mff gain‐of‐function experiments were performed in CMECs from Mff^gt^ using adenovirus vector (Ad+Mff^gt^ group). Meanwhile, Mdivi‐1, an inhibitor of fission, was used in CMECs from WT mice as the negative control group. The ischemia/reperfusion (IR) injury in vitro was mimicked by 30 minutes of hypoxia with serum starvation and 2 hours of reoxygeneration. A and B, Mitochondria of CMECs are labeled with anti‐Tom20 antibody to determine the number of cells with mitochondria fragmentation. The boxed area under each micrograph is enlarged to determine mitochondria fragmentation. To assess changes in mitochondrial morphology quantitatively, the aspect ratio (AR; the mitochondrial length) and form factor (FF; the degree of mitochondrial branching) were calculated for each cell (the minimum value for both parameters is 1). High FF and AR values show healthy mitochondria, whereas low FF and AR indicate fragmented mitochondria. C, Co‐localization of dynamin‐related protein 1 (Drp1) and mitochondria. The boxed area under each micrograph represents the amplification of the white square. More Drp1 was located on fragmented mitochondria while loss of Mff could reduce Drp1 migration on mitochondria. D through F, IR increased mitochondria‐Drp1 expression. Meanwhile, IR also reduced proteins related to mitochondrial fusion. The control of cytoplasm and mitochondrial fractionation in the Western blots are β‐actin and cytochrome c oxidase subunit IV, respectively. D and G, Caspase‐3 activation (CL.caspase3 expression) was detected by Western blots. H, Mff and CL.caspase3 co‐location by immunofluorescence. I, Transendothelial electrical resistance (TER) and permeability examination in CMECs subjected to IR injury. Confluence of CMECs monolayer was assessed as stabilized basal resistance of 800 Ω. TER increases when endothelial cells adhere and spread out, and decreases when endothelial cells retract or lose adhesion, which is the marker of endothelial barrier function. J, Fluorescein isothiocyanate (FITC)–dextran clearance was measured to assess changes in endothelial permeability. FITC‐dextran was added on top of the inserts, allowing it to permeate through the cell monolayer. The increased endothelial permeability could retain more FITC‐dextran. Thus, the FITC content remaining on the plate after IR injury indicated the extent of permeability of CMECs. *P<0.05 vs the control group; ^#^ *P*<0.05 vs the WT group; ^&^ *P*<0.05 vs the Mff^gt^ group.

To further investigate the damaging effects of Mff in microvascular IR injury, CMECs were obtained from Mff^gt^ and WT mice, and the identification of CMECs was conducted via CD31 staining and the Dil‐acetylated low‐density lipoprotein intake phagocytic test (Figure S3A and S3B). Then, 30 minutes of hypoxia with serum starvation and 2 hours of reoxygenation was used to mimic the IR injury in vitro. Owing to Mff is localized to the surface of the outer mitochondrial membrane, where it recruits cytoplasmic Drp1 to mitochondria and initiates mitochondrial fission. Therefore, we observed the changes in mitochondrial morphology under IR injury without Mff. The mitochondria in Mff^gt^ cells or Mff knockdown cells had fewer free ends than those in the IR injury group (Figure 4A and 4B). Many bulblike structures were observed at the base of the mitochondrial tubules in Mff^gt^ cells but not in the cells of the IR injury group. We also used Mdivi‐1, an inhibitor of mitochondrial fission that disrupts the Drp1‐mitochondria interaction, in WT CMECs as the negative control group.^17^ Mdivi‐1 reduced mitochondrial fragmentation and swelling, thereby preserving the mitochondrial network morphology. Drp1 shuttling between the cytoplasm and the mitochondrial surface is indispensable to mitochondrial fission. Thus, we assessed the changes in the subcellular location of Drp1 via immunofluorescence and Western blots. Figure 4C shows that IR significantly increased the overlap of Drp1 and mitochondria when compared with the control group. The results also demonstrated that the mitochondria marked by Drp1 had greater amounts of free debris. However, in Mff‐silenced WT cells or Mff^gt^ cells, Drp1 foci on the mitochondria was clearly decreased, and the mitochondria maintained almost normal morphology with fewer fragments. Mdivi‐1 could also suppress the translocation of Drp1 to the mitochondria, thereby reducing fission. In addition to fission‐related proteins, we also found that IR injury obviously reduced the expression of proteins related to mitochondrial fusion, including mitofusin 1 and mitofusin 2 (Figure 4D through 4F). These data suggested that IR injury induced the imbalance of mitochondrial dynamics, leading to excessive mitochondrial fission via Mff reupregulation and subsequent Drp1 recruitment to the mitochondria. Next, to establish the role of Mff‐mediated fission in CMEC damage, the activation of caspase and the co‐localization of CL.caspase3 and Mff were detected. Compared with the control group, IR substantially activated caspase3 (Figure 4D and 4G) and also increased the contents of CL.caspase3 and Mff (Figure 4H). However, the loss of Mff (WT+RNAi_‐Mff_ group and Mff^gt^ group) could obviously reduce the expression of CL.caspase3 and their co‐location, suggesting that Mff expression tended to be similarly correlated with that of activated caspase‐3 (Figure 4H). To examine the effect of IR injury on endothelial barrier and permeability, the changes of TER and FITC‐dextran clearance in response to IR injury were monitored (Figure 4I and 4J). Compared with the control group, IR injury significantly reduced the TER value but increased the FITC‐dextran contents, which were reversed by the loss of Mff or Mdivi‐1. These data indicated that Mff‐mediated fission was responsible for the damage to CMEC barrier function and permeability resulting from IR injury.

### Mff‐Mediated Fission Contributes to the Disruption of Mitochondrial Structure and Function

**Figure 5:**
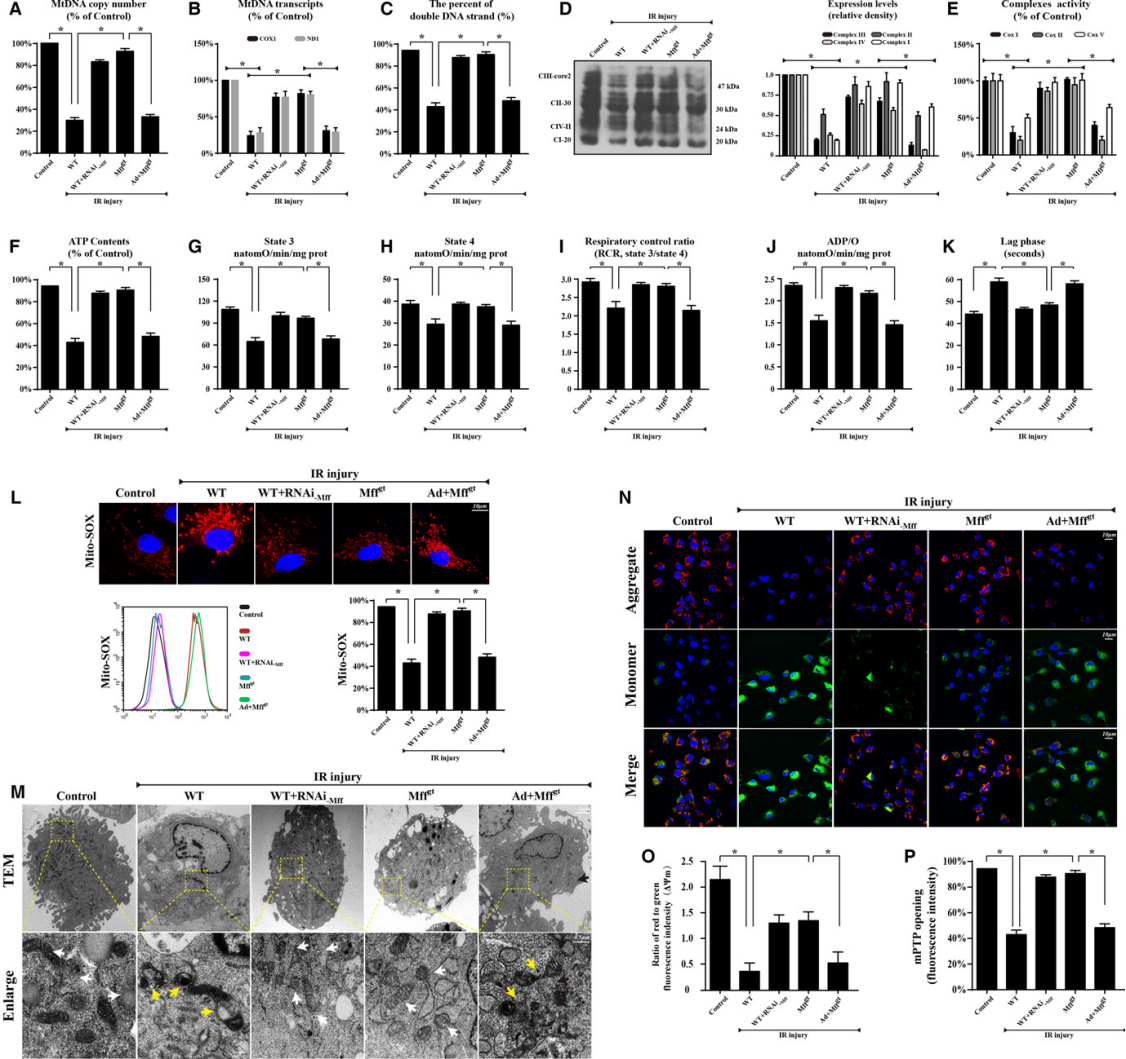
Loss of mitochondrial fission factor (Mff) protected mitochondrial structure and function against ischemia/reperfusion (IR) injury. A, mtDNA copy number was assessed by complex IV segment. B, The transcript level of mtDNA was reflected by two different components: NADH dehydrogenase subunit 1 (ND1) encoded by the light chain of mtDNA and cytochrome c oxidase subunit I (COX I) encoded by the heavy chain of mtDNA. C, The percentage of double‐stranded mtDNA indicated the mtDNA strand breaks. D, The expression of mitochondrial electron transport chain complexes (ETCx). Complex III subunit core 2, 47 kDa—CIII‐core2”; complex II‐FeS subunit, 30 kDa—“CII‐30”; complex IV subunit II, 24 kDa—“CIV‐II”; complex I subunit NDUFB8, 20 kDa—“CI‐20”. E, Changes in ETCx I, II, and V activities measured spectrophotometrically. F, Change in ATP contents. G through K, Effect of Mff‐mediated fission on state 3 respiration, state 4 respiration, respiratory control ratio (RCR [state 3/state 4]), number of nmol ADP phosphorylated to atoms of oxygen consumed (ADP/O), and ADP phosphorylation lag phase (time elapsed in the depolarization/repolarization cycle during ADP phosphorylation). L, Mitochondrial reactive oxygen species contents. The curve chart indicates the quantitative flow cytometry results. M, Representative transmission electron microscopic (TEM) images of morphological changes in mitochondria in CMECs. White arrow: normal mitochondria that exhibit a spindle shape. Yellow arrow: the divisive mitochondria. N and O, Loss of Mff could preserve mitochondrial membrane potential (∆Ψm) by JC‐1 staining. P, Change in mitochondrial permeability transition pore (mPTP) opening. *P<0.05. Mff^gt^ indicates homozygous Mff–deficient mice; WT, wild‐type.

To further determine how fission induces CMEC death, we focused on the changes to mitochondrial structure and function. As a consequence of Mff‐mediated fission, the mtDNA copy number (Figure 5A) and mtDNA transcripts (Figure 5B) were downregulated, but the loss of Mff reversed these changes. Furthermore, the impairment of replication and transcription may be attributed to the breakage of mtDNA followed by fission because IR injury reduced the amount of double‐stranded mtDNA (Figure 5C); the loss of Mff restored the content of double‐stranded mtDNA. Because the electron transport chain complexes (ETCx) are mainly encoded by mtDNA, Mff‐associated fission also reduced the content and function of ETCx (Figure 5D and 5E). ETCx‐dependent mitochondrial functions, including ATP generation, the state 3 respiratory rate, ADP phosphorylation (respiratory control ratio), and the efficiency of ATP synthesis (ADP/O), were significantly reduced following Mff‐mediated fission (Figure 5F through 5K). These changes were coupled to the increase in mROS (Figure 5L). In addition to these functional changes, fission resulted in the formation of numerous round mitochondrial fragments of varying sizes (Figure 5M). However, the Mff‐depleted cells retained a near‐normal reticulo‐tubular mitochondrial morphology during IR injury. Moreover, Mff‐mediated fission also dissipated the mitochondrial membrane potential and increased the rate of mPTP opening (Figure 5N through 5P), which was reversed by the loss of Mff.

### Mff‐Mediated Fission Facilitates HK2 Separation From the Mitochondria via the Induction of VDAC1 Oligomerization, Leading to the Opening of mPTP

**Figure 6:**
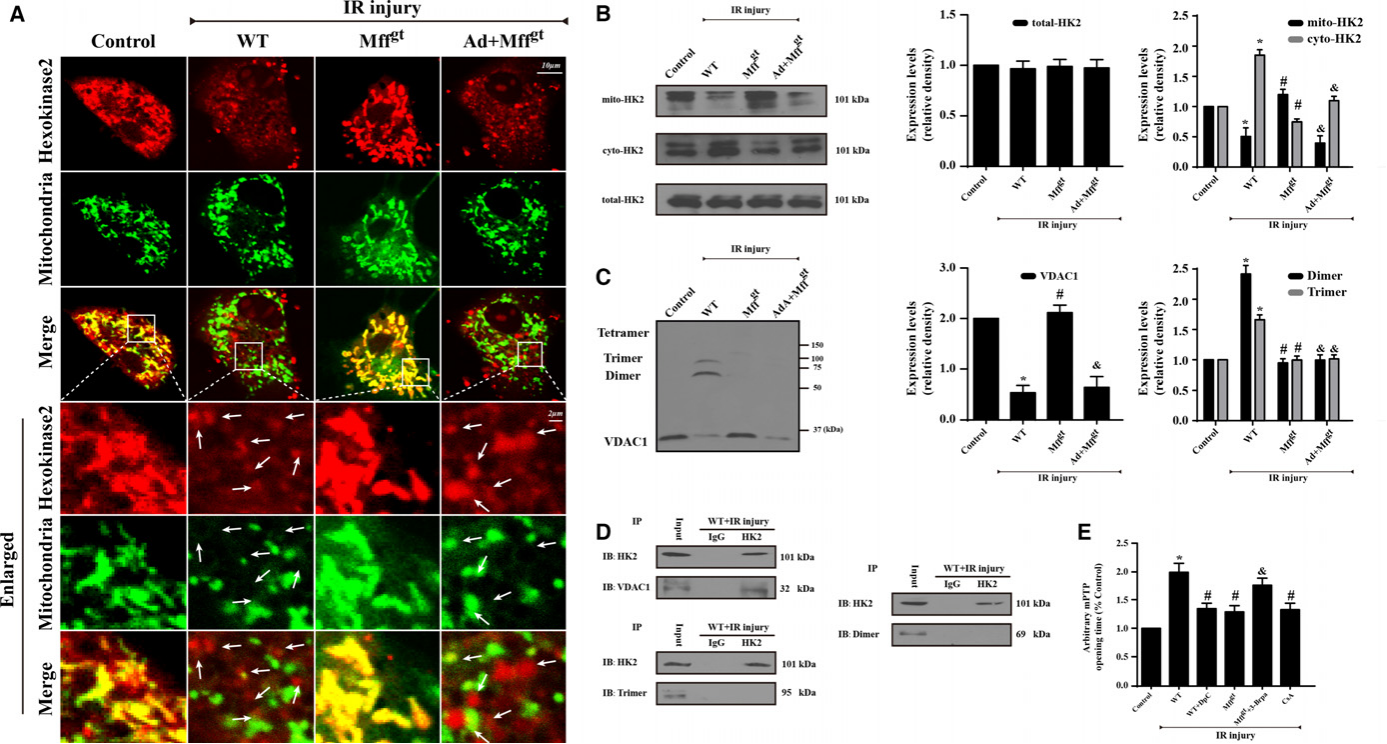
Mitochondrial fission factor (Mff) deficiency inhibits mitochondrial permeability transition pore (mPTP) opening via the reduction of voltage‐dependent anion channel 1 (VDAC1) oligomerization and subsequent hexokinase 2 (HK2) separation from the mitochondria. A and B, The subcellular location of HK2 via immunofluorescence and Western blots. Ischemia/reperfusion (IR) injury contributes to the HK2 separation from the mitochondria to the cytoplasm, which is reversed by the loss of Mff. However, IR or Mff deficiency has no effects on the total content of HK2 but influences its subcellular distribution between the mitochondria and cytoplasm. C, The evaluation of VDAC1 oligomerization via ethylene glycolbis–based cross‐linking and immunoblotting using anti‐VDAC1 antibodies. IR injury increases VDAC1 oligomerization corresponding to molecular masses of 69 and 95 kDa, whereas the loss of Mff can reverse this change. D, HK2 and VDAC1 interaction assessed by immunoprecipitation (IP) experiments. E, Arbitrary mPTP opening time by tetramethylrhodamine ethyl ester fluorescence of diphenylamine‐2‐carboxilic acid (DpC) and 3‐bromopyruvate (3‐Brpa), which are the inhibitors of VDAC1 oligomerization and HK2 interaction, respectively. Arbitrary mPTP opening time was determined as the time when the tetramethylrhodamine ethyl ester fluorescence intensity decreased by half between the initial and residual fluorescence intensity. Cyclosporin A (CsA), an mPTP blocker, was used as the negative control. *P<0.05 vs the control group; ^#^ *P*<0.05 vs the wild‐type (WT) group; ^&^ *P*<0.05 vs the Mff^gt^ group. The white arrow indicates the separated HK2 from mitochondria.

Long‐lasting mPTP opening leads abruptly to the complete dissipation of mitochondrial membrane potential and provides a channel for the leakage of mitochondrial contents, such as cyt‐c,^12^, ^26^ into the cytoplasm. The interactions of HK2 are highly protective against IR injury via the suppression of long‐lasting mPTP opening.^27^, ^28^, ^29^ Thus, to explore the mechanism by which fission induces mPTP opening, we first observed the changes in HK2. The results in Figure 6A and 6B suggest that IR injury regulates HK2 translocation between the mitochondria and cytoplasm, resulting in lower levels of mitochondrially bound HK2 but higher levels of cytoplasmic HK2. In the enlarged image, fragmented or swollen mitochondria captured less HK2 in response to IR injury, while the loss of Mff promoted HK2 remigration to the mitochondria. Interestingly, despite the decrease in mitochondrial‐related HK2, there was no change in the total content of HK2 in CMECs after IR injury (Figure 6B), suggesting that IR injury only influenced the subcellular distribution of HK2. Considering that HK directly interacts with VDAC1 to prevent mPTP opening via the suppression of the Bax/Bad interaction with VDAC1, we speculated that the dissociation of HK2 from the mitochondria was attributed to the changes in VDAC1 resulting from Mff‐mediated fission. The results suggested that IR injury significantly reduced the content of VDAC1 but increased the percentage of VDAC1 oligomerization products with molecular masses of 69 and 95 kDa (Figure 6C). To explore whether the oligomerization state of VDAC1 influenced the liberation of HK2 into the cytoplasm, we focused on protein interactions between HK2 and VDAC1 or VDAC1 dimers and multimers. Our results showed an interaction between VDAC1 and HK2 but not between oligomeric VDAC1 and HK2 (Figure 6D). These data suggested that Mff‐mediated fission caused the VDAC1 oligomerization that is responsible for the separation of HK2 from the mitochondria. To further provide evidence for the role of VDAC1 oligomerization and subsequent HK2 liberation during mPTP opening, we used diphenylamine‐2‐carboxylic acid and 3‐bromopyruvate to inhibit VDAC1 channel oligomerization and HK2 interaction, respectively. We also used Cyclosporin A, an inhibitor of mPTP opening, as the negative control group. The results indicated that inhibiting the interaction between HK2 and VDAC1 caused a longer arbitrary mPTP opening time when compared with the WT group. However, the repression of VDAC1 oligomerization shortened the arbitrary mPTP opening time (Figure 6E). These results indicated that Mff‐mediated fission facilitated the separation of HK2 from mitochondria via VDAC1 oligomerization, thereby contributing to mPTP opening.

### Mff‐Mediated Fission Aggravates cyt‐c Release via the Oxidation of CL and Activates Caspase‐9–Mediated Mitochondrial Death Pathways

**Figure 7:**
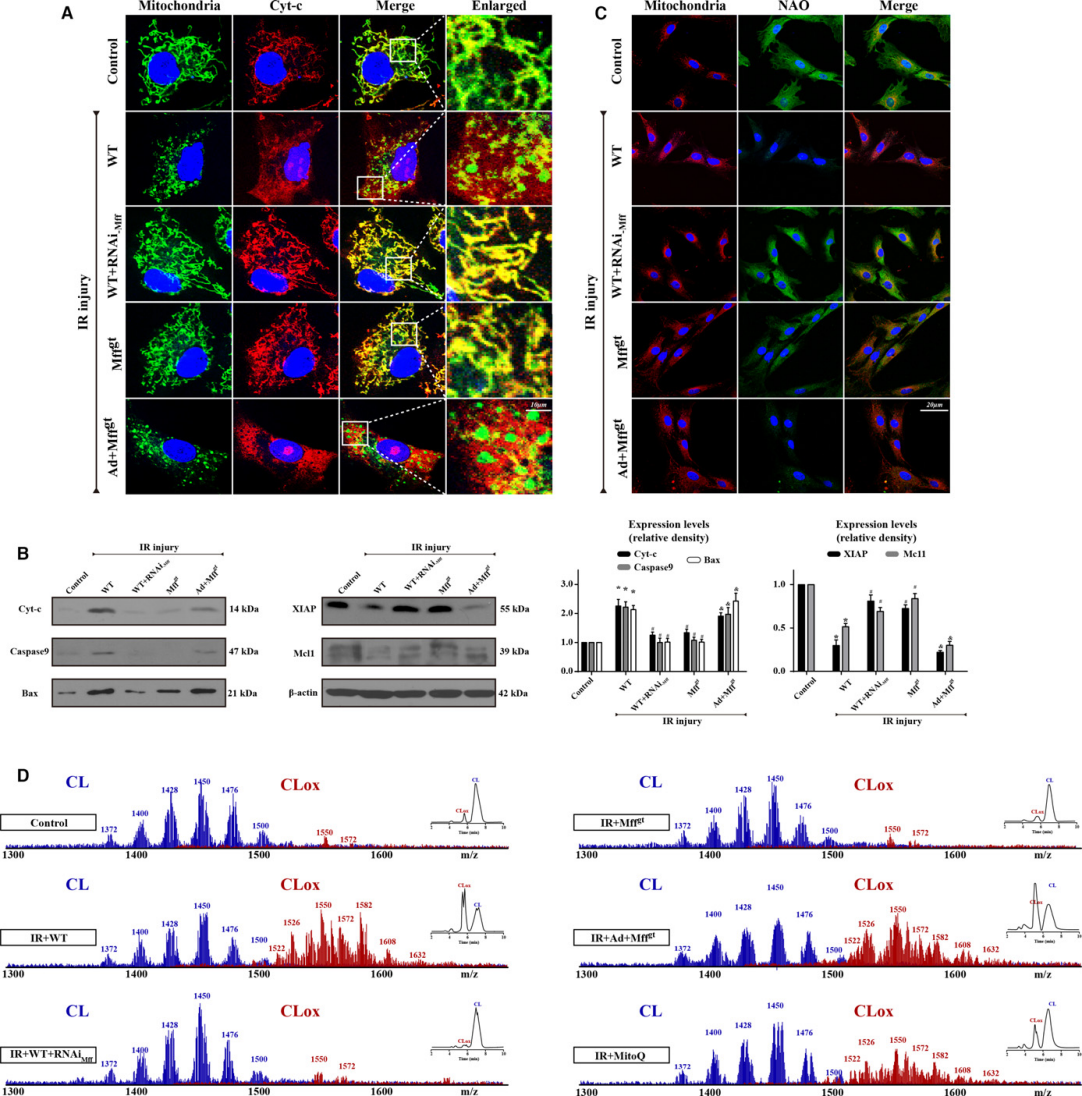
Mitochondrial fission factor (Mff)–mediated mitochondrial fission aggravates cyt‐c release via the oxidation of cardiolipin, which activates caspase‐9–involved mitochondrial death pathways. A, Immunostaining of cyt‐c leakage from mitochondria into cytoplasm. B, Changes in protein expression in association with mitochondrial apoptosis pathways. C, The changes in 10‐N‐nonyl acridine orange (NAO) fluorescence indicated ischemia/reperfusion (IR) injury induced cardiolipin (CL) oxidation. D, Assessment of molecular species of CL and its oxidation products. Left panel indicates the nonoxidized (blue) and the appearance of numerous oxidized (red) CL species after IR injury. Right inserts: 2‐dimensional chromatographic separation of nonoxidized and oxidized CL (CLox). IR injury oxidated CL via mitochondrial reactive oxygen species overproduction. *P<0.05 vs the control group; ^#^ *P*<0.05 vs the wild‐type (WT) group, ^&^ *P*<0.05 vs the homozygous Mff‐deficient (Mff^gt^) group.

After mPTP opening, cyt‐c, a subunit of oxidative phosphorylation, detaches from the mitochondrial inner membrane and leaks into the cytoplasm, where it functions as a fatal activator of cellular apoptosis. Many studies have demonstrated that fission contributes to cyt‐c release into the cytoplasm^8^, ^30^ Our data in Figure 7A are in accordance with these findings. Both nuclear and cytosolic diffusion of cyt‐c occurred in the IR injury group when compared with the puncta shape in the control groups. Moreover, Mff silencing weakened these changes (Figure 7A). Following cyt‐c release, cells progressed toward suicide via caspase‐9–dependent pathways. As shown in Figure 7B, fission caused significant increases in the expression of proapoptotic proteins (cyt‐c, caspase‐9, and Bax) and decreased the expression of antiapoptotic proteins (X‐linked inhibitor of apoptosis protein and myeloid cell leukemia 1). However, the loss of Mff restored the balance of proapoptotic and antiapoptotic proteins.

In healthy cells, cyt‐c is preferentially bound to the inner mitochondrial membrane by an association with the anionic phospholipid CL, and cyt‐c may be reversibly liberated upon the peroxidation of CL.^31^ Thus, we reasoned that CL peroxidation may be an important pathogenic pathway in fission‐mediated cyt‐c leakage. First, we used NAO, a fluorescent probe that binds specially to CL but not the product of CL peroxidation, to obverse the changes in CL. Figure 7C shows that fission significantly induced the loss of NAO fluorescence, which was increased in Mff^gt^ groups. Because fission acts as a trigger of mROS overproduction, we assumed that the CL oxidation was derived from a fission‐related burst of mROS. Thus, we used mitoquinone, a scavenger of mROS, which significantly increased the NAO fluorescence. Using 2‐dimensional high‐performance thin‐layer chromatography, we performed a global lipidomics analysis of CL (Figure 7D), which demonstrated ≈190 individual molecular species of CL in CMEC mitochondria, of which only 10 were oxygenated. Notably, fission induced the oxidation of the majority of the polyunsaturated molecular species of CL; the number of nonoxidized CL species decreased to ≈94, whereas the number of oxygenated species increased to ≈169 (Figure S4). However, Mff knockdown decreased the number and content of oxidized molecular species, which was similar to the results in the mitoquinone groups. These data suggest that fission promoted CL peroxidation.

## Discussion

**Figure 8:**
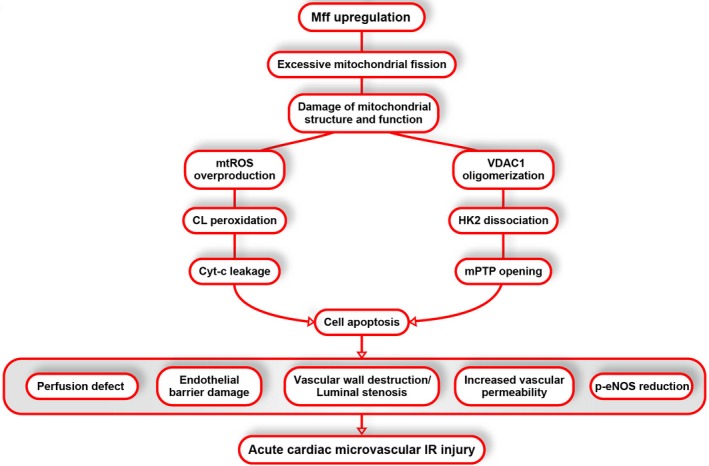
Ischemia/reperfusion (IR)–mediated mitochondrial fission factor (Mff) upregulation is accompanied by increased mitochondrial fission and reduced fusion, leading to mitochondrial structural and function damage. Voltage‐dependent anion channel 1 (VDAC1) oligomerization in response to Mff‐mediated fission leads to the separation of hexokinase 2 (HK2) from the outer mitochondrial membrane due to the lower affinity between VDAC1 multimers and HK2, resulting in the opening of mitochondrial permeability transition pore (mPTP). Moreover, increased mitochondrial reactive oxygen species (mROS) induces cardiolipin (CL) peroxidation, which mediates cyt‐c release and activation of mitochondrial‐dependent apoptosis pathways. Eventually, the apoptosis of cardiac microcirculation endothelial cells (CMECs) contributed to microvascular perfusion defect, barrier damage, vascular wall destruction or luminal stenosis, increased vascular permeability, and phospho‐endothelial nitric oxide synthase (p‐eNOS) reduction that are responsible for acute cardiac microcirculatory IR injury.

Successful percutaneous coronary intervention could open the epicardial occluded vessels but also bring about IR injury to the heart. Due to the critical and essential role of microcirculation in the matter, oxygen and energy exchange between blood and cardiomyocytes, the reperfusion injury of the microvascular bed^32^ postischemia could induce NR phenomenon that augmented additional myocardial damage and increased 30‐day mortality rates.^33^ Notably, the overwhelming majority of studies have focused on the prevention and therapy directed towards the cardiomyocytes albeit with 40 years of research on IR injury.^34^ Endothelial IR injury has been the neglected component of many strategies that aim to further reduce cardiac damage after successful percutaneous coronary intervention.^35^ In the present study, we found that: (1) increased Mff expression in the microvasculature contributed to scar expansion, cardiac dysfunction, microcirculatory perfusion defects, luminal stenosis, p‐eNOS reduction, endothelial barrier damage, inflammatory cells infiltration, and CMEC death; (2) the loss of Mff attenuates excessive mitochondrial fission, which is associated with the preservation of mitochondrial function and structure; (3) Mff‐mediated fission induced HK2 dissociation from the mitochondria via the promotion of VDAC1 oligomerization, contributing to mPTP opening; and (4) Mff also triggered cyt‐c release through the peroxidation of CL via mROS overproduction, thereby activating caspase‐9–dependent cellular apoptosis. To the best of our knowledge, this is the first study to describe the role of Mff, which mediates mitochondrial fission after acute microvascular IR injury (Figure 8).

Mitochondria are dynamic organelles that not only produce ATP for cellular energy metabolism but also participate in a number of intracellular processes, such as cell division, the initiation of mitochondrial signaling pathways, cytoskeleton modulation and mobilization, the regulation of cytosolic Ca^2+^ signals and concentration, and ultimately the determination of cell survival or death. Many studies have found that during IR injury, the destruction of mitochondria caused the impairment of aerobic respiration in cardiomyocytes, leading to energy exhaustion and cellular death. However, CMECs are more dependent on glycolysis,^36^ and, therefore, energy deficiency resulting from mitochondrial injury is not the cause of microvascular IR injury. In the present study, we found that the upregulation of Mff and subsequent Mff‐mediated mitochondrial fission is responsible for microvascular IR injury. Under physiological conditions, mitochondria are capable of traversing substantial distances in the cell along microtubules by undergoing constant fission and fusion events, which enables the exchange of proteins, lipids, and mitochondrial DNA and ATP across distances within the cell. Moderate fission is essential for changes in mitochondrial abundance to meet the metabolic demands of a cell. However, excessive fission has been reported to be accompanied by cellular damage in acute cardiomyocytes following IR injury and long‐term cardiac dysfunction after acute myocardial infarction.^37^, ^38^ Previous studies have largely focused on the effects of Drp1 in fission, particularly concerning Drp1 subcellular distribution. However, little evidence indicated changes in the expression of Drp1 receptors, especially Mff. In our study, we found that increased Mff expression in response to IR injury contributed to infarction area expansion and cardiac dysfunction. Regarding the microvascular system, the upregulation of Mff was associated with obvious microcirculation perfusion defects, the cessation of blood flow, hemocyte aggregation, and damage to the CMEC structure; these effects were reversed by the depletion of Mff in Mff^gt^ mice. Furthermore, Mff deficiency could increase p‐eNOS and VE‐cadherin expression and facilitate CMEC survival. The content of p‐eNOS in CMEC is typically proportional to the lumen diameter, which changes via vasodilation to regulate the lumen patency and blood flow. The VE‐cadherin–associated barrier and CMEC integrity are the critical elements in anticoagulation. The preservation of the cellular structure and viability, the relaxation function and endothelial barrier as a result of Mff deficiency conferred the microvessels with strong resistance to acute IR injury. We conducted in vitro loss‐ and gain‐of‐function experiments showing that Mff upregulation could cause excessive fission by promoting Drp1 translocation to the mitochondria. Despite the inability of Mff to regulate the expression of cytoplasmic Drp1 or Drp1 phosphorylated activation, Mff blunted Drp1 migration to the mitochondria, thereby suppressing fission. These findings strongly highlight neglected critical information regarding fission. Although Mff is increased in response to microvascular IR injury, this receptor has been substantially underestimated by many reports when compared with Drp1. The consequence of Mff‐mediated fission is microvascular death, especially via mitochondria‐dependent apoptosis attributed to cyt‐c release and mPTP opening.^37^ Although most previous studies have established the unambiguous role of fission in triggering mitochondrial apoptotic cell death, the underlying mechanism by which fission contributed to mPTP opening and cyt‐c leakage was still unclear.

The mPTP is a multiprotein complex that opens in either a transient, low‐conductance mode that may be protective,^39^ or a long‐lasting, high‐conductance mode that eventually causes irreversible damage to the mitochondria and promotes cell death signaling via (1) an abrupt complete dissipation of mitochondrial membrane potential and (2) the release of proapoptotic proteins such as cyt‐c. The opening of mPTP can be inhibited by HK2^40^ binding to mitochondria via structural interference, with the ability of proapoptotic proteins (Bax and Bad) to translocate from the cytoplasm to mitochondria.^12^ However, we found that Mff‐mediated fission could contribute to the dissociation of HK2 from mitochondria, leading to the excessive opening of mPTP. Interestingly, there was no change in the total expression of HK2 in CMEC under IR injury. Therefore, it is reasonable to hypothesize that the decrease in the content of mitochondrial‐HK2 is mediated by its low affinity with mitochondria. It has been shown that HK2 binds to the outer mitochondrial membrane^41^, ^42^ via VDAC1, which is dynamically oligomerized.^43^ However, Mff‐mediated fission can contribute to VDAC1 oligomerization; VDAC1 oligomers show a lower affinity with HK2, resulting in the separation of HK2 from the mitochondria. However, the exact molecular mechanisms by which fission modifies VDAC1 oligomerization remain unclear, and further studies are required to clarify this point.

In normal cells, cyt‐c is trapped tightly within the inner mitochondrial membrane by nonoxidized CL. However, CL liberates cyt‐c out of the mitochondria via mPTP once CL itself is oxidized. Because CL is proximal to the respiratory chain, it is vulnerable to peroxidation. Our studies indicated that fission significantly increases the percentage of peroxidated CL, which may be a result of mROS overproduction because the removal of mROS partially reduces CL peroxidation. The outburst of mROS is the consequence of fission‐mediated mtDNA damage and respiratory chain destruction. mtDNA comprises a naked circular strand of DNA of ≈16.5 kb.^44^ It contains two promoters, a heavy strand and a light strand, and encodes 13 polypeptides involved in oxidative phosphorylation.^45^ Once mtDNA is damaged, the encoding of critical proteins for ETCx becomes deficient. Due to the flow of electrons down the ETCx to form mitochondrial membrane potential, the decrease in the content and activity of ETCx ultimately induces more electrons to prematurely react with oxygen, especially at complexes I and III; these reactions form superoxides and other types of reactive oxygen species, which are collectively known as mROS.^46^ Mff‐dependent fission caused mtDNA double‐strand breaks and suppressed mtDNA replication and transcription, which was followed by a steep decrease in the content and function of ETCx that contributed to the overproduction of mROS. These findings address the gaps in understanding of how Mff‐dependent fission induces cyt‐c leakage into the cytoplasm; this effect occurs through CL peroxidation.

### Study Limitations

There are some limitations in the present study. First, the present study especially explored the mechanism by which IR induced cardiac microvascular injury. Whether the present findings can be applied to cardiomyocytes IR injury warrants further investigation. Second, our experiments were conducted in global Mff knockout mice. The effects of endothelial cell‐specific Mff deletion will provide further information on Mff in microvascular IR injury.

## Conclusions

We described the pathological course of cardiac microvascular IR injury. During these processes, the increase in Mff‐mediated fission triggered mitochondrial apoptosis in CMECs and damaged cardiac microvascular structure and function. These findings support the conclusion that fission may be an attractive therapeutic target to modulate the resistance of the microcirculatory system to acute reperfusion damage.

## Sources of Funding

This study was supported by grants from the National Natural Science Foundation of China (No. 81030002, 81270186, 81441008, 81102079) and the science technological innovation nursery fund of People Liberation Army General Hospital (No. 16KMZ02). The funders had no role in the study design, data collection and analysis, decision to publish, or preparation of the manuscript.

## Disclosures

All authors have completed and submitted the ICMJE Form for Disclosure of Potential Conflicts of Interest. The authors have declared that they have no conflicts of interest.

## Supporting information


**Figure S1.** Schematic depicting gene trap insert in Mff locus. In briefly, the exon/intron structure is derived from Ensembl (transcript ID: ENSMUST00000078332). Exon 3 is the first coding exon, and exon 9 encodes the transmembrane (TM) segment. The gene trap vector pGT01xr with splice acceptor (SA) and lacZ/neomycin phosophotransferase fusion gene (β‐geo) is inserted immediately after the sequence 5′‐GCACTCCTCTGTCTGCCTTG‐3′ in the intron following exon 4. Exon 4 encodes the Mff motifs essential for Drp1 recruitment. As a result, the gene trap insertion is ideally positioned to disrupt all Mff isoforms.
**Figure S2.** A, The transfection of Ad‐Mff in CMEC. B, Western blots were used to assess the Mff overexpression under Mffgt by Ad‐Mff.
**Figure S3.** A, CD31 immunocytochemistry of CMECs. B, Dil‐acetylated low‐density lipoprotein intake assay. Green, CD31; red, Dil‐ldl.
**Figure S4.** Evaluation of the number of nonoxidized and oxidized molecular species of CL in CMEC.
