# Supplementary material for: Mff‐Dependent Mitochondrial Fission Contributes to the Pathogenesis of Cardiac Microvasculature Ischemia/Reperfusion Injury via Induction of mROS‐Mediated Cardiolipin Oxidation and HK2/VDAC1 Disassociation‐Involved mPTP Opening
Source: J Am Heart Assoc. 2017 Mar 13;6(3):e005328. doi: 10.1161/JAHA.116.005328 (PMC5524036; doi:10.1161/JAHA.116.005328)
Supplement: Supplementary file 1 — Figure S1. Schematic depicting gene trap insert in Mff locus. In briefly, the exon/intron structure is derived from Ensembl (transcript ID: ENSMUST00000078332). Exon 3 is the first coding exon, and exon 9 encodes the transmembrane (TM) segment. The gene trap vector pGT01xr with splice acceptor (SA) and lacZ/neomycin phosophotransferase fusion gene (β‐geo) is inserted immediately after the sequence 5′‐GCACTCCTCTGTCTGCCTTG‐3′ in the intron following exon 4. Exon 4 encodes the Mff motifs essential for Drp1 recruitment. As a result, the gene trap insertion is ideally positioned to disrupt all Mff isoforms. Figure S2. A, The transfection of Ad‐Mff in CMEC. B, Western blots were used to assess the Mff overexpression under Mffgt by Ad‐Mff. Figure S3. A, CD31 immunocytochemistry of CMECs. B, Dil‐acetylated low‐density lipoprotein intake assay. Green, CD31; red, Dil‐ldl. Figure S4. Evaluation of the number of nonoxidized and oxidized molecular species of CL in CMEC. [file JAH3-6-e005328-s001.pdf]

SUPPLEMENTAL MATERIAL

Figure S1.

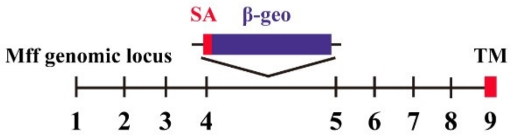

Figure S2.

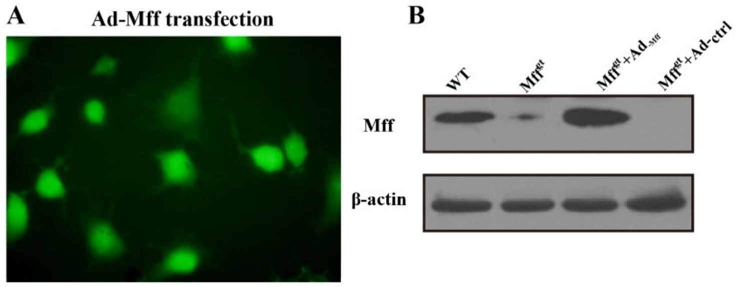

Figure S3.

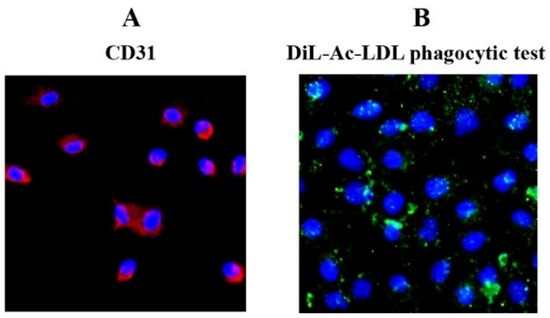

Figure S4.

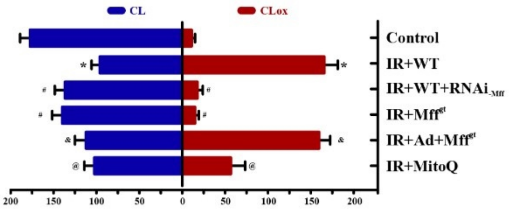

Supplemental Figure Legends:

Figure S1. Schematic depicting gene trap insert in Mff locus. In briefly, the exon/intron structure is derived from Ensembl (transcript ID: ENSMUST00000078332). Exon 3 is the first coding exon, and exon 9 encodes the transmembrane (TM) segment. The gene trap vector pGT01xr with splice acceptor (SA) and lacZ/neomycin phosophotransferase fusion gene (β-geo) is inserted immediately after the sequence 5′-GCACTCCTCTGTCTGCCTTG-3′ in the intron following exon 4. Exon 4 encodes the Mff motifs essential for Drp1 recruitment. As a result, the gene trap insertion is ideally positioned to disrupt all Mff isoforms.

Figure S2. A. The transfection of Ad-Mff in CMEC. **B.** Western blots were used to assess the Mff overexpression under Mff^{gt} by Ad-Mff.

Figure S3. A. CD31 immunocytochemistry of CMECs. **B.** Dil-acetylated low-density lipoprotein intake assay. green, CD31; red, Dil-ldl.

Figure S4. Evaluation of the number of non-oxidized and oxidized molecular species of CL in CMEC.
